# Hommage à Nicole Léger (1933-2024)

**DOI:** 10.48327/mtsi.v5i4.2025.728

**Published:** 2025-07-29

**Authors:** Jérôme DEPAQUIT, Marie-Claude DURETTE-DESSET, Jean-Charles GANTIER, Frédérick GAY, Marc GENTILINI, Nabil HADDAD, René HOUIN, Mireille KILLICK-KENDRICK, Irène LANDAU, Bernard PESSON, François RODHAIN

**Affiliations:** 1. Faculté de pharmacie, Université de Reims Champagne Ardenne, UR ESCAPE-USC ANSES PETARD, 51 Rue Cognacq-Jay, 51100 Reims, France; 2. Centre hospitalo-universitaire, Pôle de biologie territoriale, Laboratoire de parasitologie-mycologie, Reims, France; 3. Institut de systématique, évolution, biodiversité (ISYEB), Muséum national d’Histoire naturelle, CNRS, Sorbonne Université, EPHE, Université des Antilles, Paris, France; 4. Faculté de Pharmacie, Université de Paris XI, Châtenay Malabry, France; 5. Laboratoire des identifications fongiques et entomoparasitologiques, 4 rue des Sémailles, 91540 Mennecy, France; 6. Sorbonne Université, INSERM, Institut Pierre Louis d’épidémiologie et de santé publique, Hôpital Pitié Salpêtrière, AP-HP, 75013, Paris, France; 7. Organisation PanAfricaine de Lutte pour la Santé (OPALS), Paris, France; 8. Service des maladies infectieuses, Hôpital Pitié-Salpêtrière, Paris, France; 9. Laboratoire d’immunologie et de maladies à transmission vectorielle, Faculté de Santé Publique, Université Libanaise, Boîte postale 6573/14 Badaro, Musée, Beyrouth, Liban; 10. Division of Health Professions, Faculty of Health Sciences, American University of Beirut, Beyrouth, Liban; 11. Laboratoire de parasitologie-mycologie, Hôpital Henri Mondor AP-HP, Université Paris XII, 1 Rue Gustave Eiffel, 94000 Créteil, France; 12. Imperial College at Silwood Park, Buckhurst Rd, Berks SL5 7PY, Royaume-Uni; 13. Molécules de communication et adaptation des microorganismes, UMR 7245. Muséum national d’Histoire naturelle, Paris; 14. Laboratoire de parasitologie, faculté de pharmacie, Université de Strasbourg, 72 route du Rhin, CS 10315,67411 Illkirch-Graffenstaden Cedex, France; 15. Institut Pasteur, 25 Rue du Dr Roux, 75015 Paris, France

**Figure 1:**
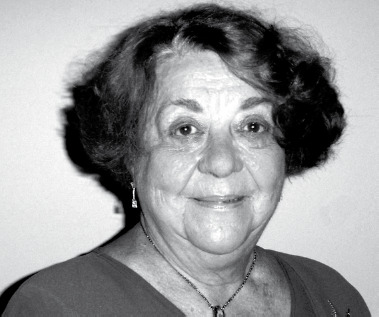
Portrait de Nicole Léger pris au début des années 2000 (crédit photo: Bob Killick-Kendrick)

Née en 1933, Nicole Léger accomplit ses études de pharmacie de 1950 à 1955 à la faculté de Paris et complète son diplôme en 1956 et 1957 avec les certificats supérieurs spéciaux de chimie biologique, de parasitologie, de sérologie et de bactériologie. Reçue interne des hôpitaux de Paris en 1954, puis interne des hôpitaux psychiatriques en 1958, Nicole Léger entre en 1959 à la faculté de pharmacie de Paris comme assistante aux travaux pratiques de parasitologie dirigés par le professeur Cavier. Elle devient chef de travaux, puis maître-assistante, assurant successivement l’enseignement au certificat supérieur spécial de parasitologie, aux travaux pratiques de zoologie de première année, aux enseignements dirigés de quatrième année, aux travaux pratiques de cinquième année, suivant en quelque sorte la progression de la réforme des études pharmaceutiques et mettant sur pied, chaque année, un enseignement nouveau.

Dans le même temps, elle prépare et soutient une licence en sciences et présente en 1965 une thèse de doctorat ès sciences pharmaceutiques (diplôme d’État) intitulée « Contribution à l’étude du comportement biologique du cysticercoïde *dHymenolepis nana* var. *fraterna* vis-à-vis de ses hôtes et des médicaments », pour laquelle elle a été reçue avec la mention très honorable, et a obtenu le prix de thèse, mention sciences naturelles.

À partir de 1963, elle a assuré les fonctions d’attachée à l’Assistance Publique, d’abord à l’hôpital Corentin Celton en tant qu’attachée de parasitologie et, à partir de 1968, dans le service du professeur Marc Gentilini à l’hôpital Saint-Louis puis à l’hôpital de la Pitié-Salpêtrière. Reçue à l’agrégation de médecine, elle se présente alors à celle de pharmacie où elle sera reçue. Nommée professeur à Reims en 1972, elle y terminera sa carrière officielle en 2001.

Du fait de ses activités variées, ses travaux de recherche axés au départ sur la pharmacodynamie des anthelminthiques se sont peu à peu diversifiés. L’idée directrice de sa thèse était qu’au cours de leur cycle, les helminthes passaient par une suite de révolutions biologiques et que chaque stade correspondait à une sensibilité différente aux agents extérieurs, et en particulier aux médicaments employés en chimiothérapie, si bien que ceux-ci pouvaient être totalement inactifs sur certains de ces stades. La résistance des formes immatures aux traitements anthelminthiques était d’ailleurs bien connue. C’est ce qu’elle a pu démontrer avec le dichlorophène et la mépacrine chez le cysticercoïde *dHymenolepis nana,* aussi bien dans le cas où le cycle était direct que dans celui où il passait par un hôte intermédiaire. Elle a également essayé de préciser le mode d’action de divers ténicides sur la forme adulte du parasite. Les modifications morphologiques des helminthes sous l’effet de divers ténicides et vermifuges ont été étudiées *in vivo* chez la souris. Ce travail lui a permis de démontrer que leurs actions étaient loin d’être univoques et elle a pu individualiser au moins deux mécanismes différents.

**Figure 2:**
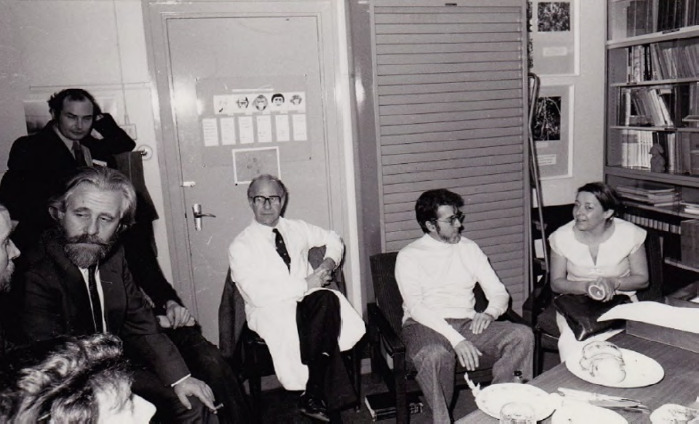
Laboratoire de parasitologie de la London School of Tropical Medicine and Hygiene montrant de gauche à droite Bob Killick-Kendrick devant David Molyneux, Wallace Peters en blouse blanche, Jean-Antoine Rioux et Nicole Léger, photo prise au début des années 1970 (crédit photo: LSTMH)

Son étude sur la réactivité de la forme larvaire *dHymenolepis* chez le ténébrion l’a conduite à étudier les relations hôte-parasite dans le système insecte-cysticercoïde. L’étude des interactions hôtes-parasites constituera d’ailleurs un intérêt scientifique majeur de l’ensemble de sa carrière. S’attachant à l’étude des conditions d’apparition des réactions au parasitisme chez les insectes, elle constate l’absence de phénomène visible chez les espèces normalement infectables par la voie orale. Les insectes réfractaires à ce mode d’infection avaient pu être parasités expérimentalement par l’injection intra-cavitaire d’embryons hexacanthes éclos *in vitro.* Les insectes réagissaient alors par encapsulation et mélanisation des parasites et Nicole Léger pensait que les cellules hémocytaires semblaient jouer un rôle essentiel. L’hémocoele des insectes, non infectables par la voie orale, constitue donc un milieu convenable pour le développement larvaire *dHymenolepis nana.* Le problème était alors de savoir à quel niveau se situait la barrière à la contamination. Nicole Léger a montré que l’éclosion de l’œuf était normale dans la lumière de l’intestin, libérant un embryon viable. En récupérant cet embryon dans les fèces et en l’injectant à un nouvel insecte, il se développe et se transforme en un cysticercoïde infectant pour la souris. Après avoir avancé l’hypothèse que c’était la membrane péritrophique qui constituait la barrière au passage de l’embryon hexacanthe et à son développement ultérieur dans la cavité générale, elle pensait, à la lumière de ses travaux d’histologie, que la destruction avait lieu aussitôt après la traversée de l’épithélium intestinal. Nicole Léger s’est également intéressée aux relations entre le ver adulte et l’hôte définitif, en collaboration avec le professeur Ambroise Thomas.

Cependant, la direction prise par ses recherches n’a pas détourné l’intérêt initial de Nicole Léger pour la pharmacodynamie des antiparasitaires. C’est ainsi que dans le cadre de ses recherches sur les relations structure chimique - activité pharmacodynamique, elle a effectué divers essais de ténicides sur *Hymenolepis nana* var. *fraterna in vitro* et *in vivo* chez la souris ainsi que de vermifuges sur *Syphacia obvelata* et *Aspiculuris tetraptera,* oxyures de la souris et sur *Nippostrongylus brasiliensis* du rat. Sa tentative d’utilisation de *Dipetalonema vitae* pour l’essai de filaricides s’est malheureusement soldée par un échec, ce parasite du mérion, adapté au hamster, s’étant révélé insensible aux médicaments utilisés dans les filarioses humaines. En revanche, *Dipetalonema vitae* s’est révélée être un modèle performant pour l’étude de l’action histopathogène, sans doute en partie de nature immunitaire, des filaires. C’est ainsi qu’elle a entrepris en collaboration avec le professeur Gentilini, l’étude des lésions rénales observées au cours des filarioses, l’équipe médicale se chargeant de la partie clinique, et Nicole Léger assurant la partie expérimentale du travail. L’entretien des souches nécessaires à ses essais lui permettait en outre de mettre à la disposition des immunologistes du laboratoire de parasitologie du CHU de la Pitié-Salpêtrière le matériel nécessaire aux travaux de routine et à la recherche: *Dipetalonema vitae, Nippostrongylus brasiliensis, Trypanosoma lewisi*, et *Pneumocystis carinii*, qu’elle fut la première en France à avoir pu obtenir expérimentalement chez le rat.

Avec *Anthemosoma garnhami,* elle pensait avoir montré que parfois, la preuve thérapeutique pouvait venir en aide au systématicien. Ce parasite, isolé en 1967, à partir de rongeurs du genre *Acomys,* et entretenu par la suite sur les souris, est un protozoaire intra-érythrocytaire non pigmenté. Il a été décrit en 1969 par Irène Landau, professeur au Muséum national d’Histoire naturelle de Paris. L’étude de sa morphologie incitait cette dernière à rattacher ce parasite, parmi les Babesioideae, à la famille des Dactylosomidae. Comme les espèces déjà décrites appartenant à cette famille sont toutes parasites de vertébrés à sang froid (batraciens, reptiles), Nicole Léger se posait la question de savoir s’il ne s’agissait pas plutôt d’un hématozoaire non pigmenté peut-être plus proche des *Plasmodium* que des *Babesia.* Elle voulait démontrer l’intérêt qu’il y aurait, pour les systématiciens, à prendre plus souvent en compte l’épreuve thérapeutique, puisque les médicaments actifs sur les *Babesia* se montrent inactifs sur les *Plasmodium* et vice-versa. Nicole Léger a donc entrepris l’étude de l’action de ces deux groupes de substances sur *A. garnhami.* Les anti-malariques testés furent choisis parmi ceux qui se montraient particulièrement actifs sur les *Plasmodium* de la souris: quinine, quinacrine, chloroquine, sulfadiazine, chlortétracycline. Aucun ne s’est montré actif sur *A. garnhami,* l’évolution de l’infestation étant la même chez les animaux traités et chez les animaux témoins. En revanche, tous les anti *Babesia* testés (acaprine, acriflavine, pentamidine) se sont montrés efficaces. Ce résultat venait donc renforcer l’opinion d’Irène Landau et la validité du nouveau genre proche des *Babesia* créé par cette dernière. Cependant, *Dactylosoma* et les Dactylosomidés se sont révélés devoir, maintenant, être classés dans les Coccidies.

L’étude des mécanismes d’action des médicaments antiparasitaires, ainsi que les relations hôtes-parasites chez des invertébrés était déjà pour Nicole Léger l’occasion d’envisager les rapports existant entre le parasite et son milieu. C’est donc tout naturellement qu’elle s’est ouverte à l’écologie parasitaire, d’autant plus que sa formation de naturaliste l’y prédisposait. Se mettant à l’école des parasitologues de terrain, elle a d’abord entrepris avec son collègue Claude Combes de Perpignan, une étude sur la répartition géographique des Trématodes de batraciens. Dans un premier travail, elle a recherché les variations dans le temps des populations d’helminthes parasites de *Rana temporaria* dans une région recouvrant la Cerdagne et une partie de l’Andorre précédemment prospectées. Elle y a constaté, au moins qualitativement, une grande stabilité de la répartition des Digènes et des Monogènes. Les seules variations observées intéressaient des stations qui avait été entre-temps profondément remaniées par l’être humain et s’expliquaient par le bouleversement des conditions écologiques, assurant le maintien des populations de mollusques hôtes intermédiaires et, à un moindre degré, des populations de grenouilles.

La seconde région que Nicole Léger a prospectée était la Camargue. Dans un biotope où vivaient en étroite cohabitation grenouilles vertes et rainettes, elle a observé, rien que chez *Hyla meridionalis,* quatre espèces de Digènes alors qu’il s’agit d’un batracien possédant jusqu’alors la réputation d’être réfractaire à l’infection par ces Trématodes. Elle a en outre entrepris l’étude de la faune helminthologique des batraciens de Corse, dont l’originalité, en rapport avec les phénomènes d’insularité, lui semblait d’ores et déjà poser de passionnantes questions de phylogénie et de phylogéographie.

Dès les années 1970, elle participe à des travaux de malacologie en Corse et conclut que tout est prêt pour l’introduction de *Schistosoma haematobium* sur l’île de beauté. Nous apprendrons dès 2013 que cette prédiction était devenue réalité. Poursuivant ce travail sur les bilharzioses, elle montrera le caractère zoonotique de la bilharziose à *Schistosoma mansoni* en Guadeloupe avec l’intervention du rat dans le cycle parasitaire. À la fin de sa carrière, elle travaillera à nouveau, avec son élève Hubert Ferté, sur les schistosomes lors de la survenue d’épisodes de dermatite cercarienne causés par des bilharzies aviaires sur de nombreux lacs français.

Mais en fait, c’est l’écologie des cycles parasitaires empruntant la voie de l’insecte qui, du fait de ses travaux antérieurs, devait retenir toute son attention. Sa rencontre avec le professeur Alain Chabaud du Muséum national d’Histoire naturelle de Paris, fut déterminante dans sa carrière, débutant une collaboration avec lui dans le cadre d’un programme d’élaboration en Camargue d’un modèle pour l’étude de l’épidémiologie des filarioses.

**Figure 3:**
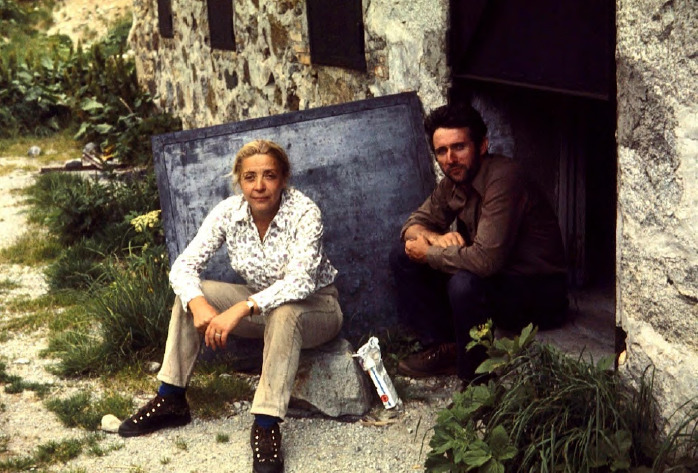
Nicole Léger et Claude Combes, sur le terrain au lac des Bouillouses (Cerdagne) en 1970 (crédit photo: Bernard Pesson)

À la survenue des premiers cas de paludisme des aéroports à la fin des années 70, le ministère de la Santé la missionna pour effectuer une prospection entomologique dans et aux abords des aéroports internationaux parisiens. Elle communiqua les résultats de son enquête lors du symposium organisé en 1980 à Strasbourg pour le centenaire de la découverte de l’agent du paludisme par Alphonse Laveran.

Une autre rencontre qui fut déterminante dans la carrière de Nicole Léger fut celle du professeur Jean-Antoine Rioux de Montpellier qui, dans le cadre de ses enquêtes épidémiologiques sur les leishmanioses en Afrique du Nord, a entrepris l’étude des phlébotomes de Corse et de leur répartition. L’utilisation de méthodes de piégeage éprouvées et pratiquées depuis longtemps lui a permis de progresser rapidement. Elle utilisait surtout la technique des pièges adhésifs non attractifs qui lui permettait d’apprécier la densité relative de chaque espèce en rapportant le nombre d’individus capturés au mètre carré de piège. En deux ans, elle a ainsi prospecté la totalité de la Corse explorant près de 400 stations et récoltant plus de 20 000 phlébotomes tous examinés un par un et identifiés à l’espèce. Ce travail a permis d’y signaler pour la première fois *Phlebotomus sergenti,* le principal vecteur de *Leishmania tropica.* Forte de cet inventaire, il lui est alors possible de se faire une idée assez précise des possibilités de transmission de la leishmaniose humaine en Corse. *Phlebotomusperniciosus* lui semble devoir y jouer le rôle de vecteur principal, tant du fait de son anthropophilie marquée et de sa forte densité dans l’étage phytogéographique recouvrant notamment les séries du chêne pubescent et du chêne vert, qui correspondent à la répartition classique des foyers d’endémie leishmanienne dans le midi de la France continentale.

Poursuivant la collaboration fructueuse avec le professeur Rioux, elle travaillera au Maroc durant cinq années à partir de 1972 en focalisant ses travaux sur l’éco-épidémiologie des leishmanioses, maladies à précellence vectorielle dont l’étude des vecteurs est essentielle. Elle y consacrera dès lors une grande partie de la seconde moitié de sa carrière. Elle travaillera dans de nombreux foyers de leishmaniose, principalement du bassin méditerranéen. Passionnée par l’entomologie, elle prendra une place très importante dans la communauté internationale des « leishmaniaques » et des « *sand flies lovers* ». À la fin des années 1980, lorsque le professeur Robert Killick-Kendrick a commencé à concevoir un congrès centré uniquement sur les phlébotomes (ISOPS: *International Symposium On Phlebotomine Sandflies),* il pensait que le pays organisateur devait être l’Italie, pays où a été décrit le premier phlébotome, *Phlebotomus papatasi,* par Scopoli en 1786. Il a donc demandé à Michele Maroli (Istituto Superiore di Sanità, Rome) d’accepter de réaliser ce congrès à Rome. Ce dernier, au début de l’aventure, était dubitatif et se demandait combien de personnes y participeraient. C’est alors que Nicole Léger avec son enthousiasme caractéristique, a apporté tout son soutien énergique à la mise en place du premier ISOPS en 1991 en collaborant avec Michele Maroli et Robert Killick-Kendrick. Ce congrès fut un énorme succès qui se perpétue toujours. Nicole Léger ne manquera aucun congrès ISOPS à l’exception de celui de 2019 organisé aux îles Galápagos en raison de soucis de santé suffisamment sérieux pour l’en dissuader. Elle organisera celui de 2016 en France, à Reims. Un vibrant hommage lui fut rendu lors de la dernière édition en septembre 2024 en Slovénie.

Passionnée de taxinomie et de systématique, elle décrira chez les phlébotomes 36 taxons nouveaux pour la science: une tribu, deux sous-tribus, trois sous-genres et 30 espèces et sous-espèces.

**Figure 4:**
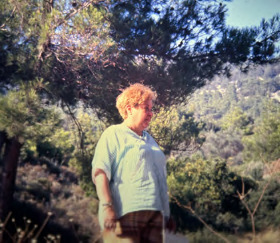
Nicole Léger sur le terrain à Rhodes en 1992 (crédit photo: Jérôme Depaquit)

**Figure 5:**
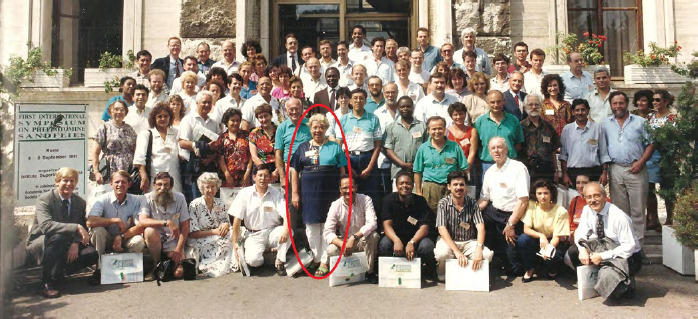
Nicole Léger au centre des participants au premier « International Symposium On *Phlebotomine Sandflies* » à Rome, en 1991 (crédit photo: ISOPS)

## Taxons décrits chez les phlébotomes par Nicole Léger

**Tribu**

Hertigiini Abonnenc & Léger, 1976

**Sous-tribus**

Hertigiina Abonnenc & Léger, 1976

Lutzomyiina Abonnenc & Léger, 1976

**Sous-genres**

*Vattieromyia* Depaquit, Léger & Robert, 2008

*Trouilletomyia* Depaquit & Léger, 2014

*Madaphlebotomus* Depaquit, Léger & Randrianambinintsoa, 2015

**Espèces et sous-espèces**

*Phlebotomus (Larroussius) mariae* Rioux, Croset, Léger & Bailly-Choumara, 1974

*Phlebotomus (Madaphlebotomus) berentiensis* (Léger & Rodhain, 1978)

*Nyssomyia pajoti* (Abonnenc, Léger & Fauran 1979)

*Psychodopygus claustrei* (Abonnenc, Léger & Fauran, 1979)

*Nyssomyia bibinae* (Léger & Abonnenc, 1988)

*Australophlebotomus maduloae* Léger & Pesson, 1993

*Australophlebotomus notteghemae* Léger & Pesson, 1993

*Idiophlebotomus boucheti* (Léger & Pesson, 1994)

*Oligodontomyia isopsi* (Léger & Ferté, 1996)

*Phlebotomus (Synphlebotomus) saltiae* Léger, Haddad & Chaker, 1997

*Phlebotomus* (*Paraphlebotomus*) *riouxi* Depaquit, Ferté & Léger, 1998

*Phlebotomus* (*Transphlebotomus*) *economidesi* Léger, Depaquit & Ferté, 2000

*Phlebotomus (Madaphlebotomus) fertei* (Depaquit, Léger & Robert, 2002)

*Sergentomyia (Trouilletomyia) huberti* Depaquit, Léger & Robert, 2002

*Phlebotomus (Madaphlebotomus) fontenillei* (Depaquit, Léger & Robert, 2004)

*Chinius barbazani* Depaquit, Léger & Beales, 2006

*Chinius eunicegalatiae* Depaquit, Léger & Beales, 2006

*Phlebotomus (Euphlebotomus) mascomai* Müller, Depaquit & Léger, 2007

*Sergentomyia majungaensis* Depaquit, Léger & Robert, 2007

*Sergentomyia (Vattieromyia) anka* Depaquit, Léger & Robert, 2008

*Sergentomyia (Vattieromyia) namo* Depaquit, Léger & Robert, 2008

*Sergentomyia (Vattieromyia) sclerosiphon* Depaquit, Léger & Robert, 2008

*Phlebotomus (Euphlebotomus) barguesae* Depaquit, Muller & Léger, 2009

*Chinius samarensis* Léger, Depaquit & Gay, 2012

*Sergentomyia (Vattieromyia) pessoni* Depaquit, Randrianambinintsoa & Léger, 2012

*Sergentomyia (Rondanomyia) goodmani comorensis* Depaquit, Randrianambinintsoa & Léger, 2012

*Phlebotomus (Madaphlebotomus) vaomalalae* (Randrianambinintsoa, Léger, Robert & Depaquit, 2013)

*Idiophlebotomus padillarum* Léger, Depaquit & Gay, 2014

*Sergentomyia brunhesi* Léger, Randrianambinintsoa & Depaquit, 2020

*Sergentomyia vistellei* Depaquit, Randrianambisintsoa & Léger, 2020

En systématique, grande admiratrice du travail de sa collègue brésilienne Eunice Galati avec laquelle elle noua une profonde amitié, Nicole Léger initia avec Philippe Rispail l’analyse phylogénétique des phlébotomes de l’Ancien Monde par analyse des caractères morphologiques. Elle fut pionnière en encadrant Jérôme Depaquit avec Guillaume Lecointre pour réaliser le premier travail de systématique moléculaire des phlébotomes. D’un enthousiasme particulièrement communicatif, elle a formé beaucoup d’élèves et a travaillé dans de nombreuses régions du monde. Elle appréciait beaucoup la Grèce où elle a réalisé un programme d’étude de la spéciation des phlébotomes en fonction de l’insularité. Infatigable voyageuse, prônant l’excellence du travail de terrain, elle participa puis dirigea de nombreuses missions dans tout le bassin méditerranéen (Corse, Espagne, Italie, Malte, Serbie, Monténégro, Grèce, Chypre, Syrie, Liban, Maroc), aux îles Canaries, en Afrique (Bénin, Cameroun, République centrafricaine, Namibie, Afrique du Sud, Tanzanie), ainsi qu’en Inde.

Dans le cadre des études sur les phlébotomes, Nicole Léger rencontra Émile Abonnenc spécialiste français incontesté de ces insectes. Elle devient une de ses élèves et reconnaît une dette immense envers lui. Elle gardera un souvenir admiratif non seulement du maître mais aussi du fidèle ami qu’il était devenu au fil des ans. Elle reconnaissait en lui un travailleur acharné, scrupuleux, doté d’une grande honnêteté ainsi que d’une rare bonté et d’un total désintéressement. En Guyane française, elle fit avec lui un immense travail de synthèse sur les phlébotomes américains. Elle était devenue si proche d’Émile Abonnenc qu’il lui avait légué sa collection de phlébotomes. Sa collection, dont elle avait hérité, et la sienne, sont désormais à la faculté de pharmacie de Reims où, comme elle le souhaitait, elles peuvent être examinées ou prêtées à la communauté des chercheurs.

Durant toute sa carrière, elle a bâti un impressionnant réseau de collaborateurs dans le monde entier comme en France dont ses élèves profitent encore.

Signe d’une large reconnaissance par ses pairs, six taxons lui ont été dédiés: un sous-genre et cinq espèces.

## Taxons dédiés à Nicole Léger

**Insectes**

Diptera, Keratoplatidae:

*Xenoplatyura nicolae* Matile, 1997

Diptera, Psychodidae:

*Lutzomyia (Tricholateralis) legerae* Le Pont, Gantier, Hue & Valle, 1995

Sous-genre *(Legeromyia)* Rahola, Depaquit, Makanga & Paupy, 2013

*Idiophlebotomus nicolegerae* Loyer, Depaquit & Gay, 2016

**Nématodes**

Nematoda, Molineidae:

*Molineus legerae* Durette-Desset & Pesson, 1987

Nematoda, Capillariidae:

*Capillaria legerae* Justine, Ferté & Bain, 1987

L’activité scientifique de Nicole Léger semble montrer une apparente hétérogénéité de l’ensemble de ses travaux. Cependant, lorsque l’on cherche à définir ses motivations profondes, on trouve toujours en elle le désir d’appréhender les facteurs réglant l’apparition et le maintien du parasitisme, que ce soit dans le cadre limité du milieu intérieur de l’hôte, ou dans celui plus vaste des biocénoses au sein desquelles les cycles se déroulent.

À une époque où, dans la plupart des sciences, une stricte spécialisation est devenue nécessité pour progresser, elle avait choisi probablement l’une des dernières disciplines où l’éclectisme des connaissances et des curiosités conservait encore toute sa valeur. En effet, le parasite est sous la stricte dépendance de son environnement et son étude ne peut être abordée sans tenir compte de celui-ci, ce qui nécessite de pouvoir être à l’occasion zoologiste, botaniste, histologiste, immunologiste, écologue, voire même chimiste. Les dernières décennies ont souvent méconnu ceux qui se voulaient naturalistes. Mais il semble désormais évident que l’on ne peut se couper de la nature sans en subir les conséquences, tant on en fait partie intégrante. De fait, l’actualité donne particulièrement raison à Nicole Léger, la réhabilitation étant finalement en cours. Qui aujourd’hui ne s’inscrit pas dans une démarche intégrant la santé humaine, la santé animale et la santé environnementale, sous le vocable de concept « une seule santé »? Elle fut finalement pionnière dans ce domaine comme dans tant d’autres.

Tout au long de sa vie et de sa carrière, Nicole Léger a eu la chance d’avoir les encouragements, l’aide et le soutien actif de son mari Pierre, chirurgien renommé. Il l’a souvent accompagnée dans les congrès et au cours de ses missions sur le terrain où il participait au travail, apportant une contribution significative aux études de son épouse. Toujours prête à vivre l’aventure, Nicole Léger n’hésitait pas à dormir à la belle étoile, dans des conditions parfois inconfortables, pour être, dès l’aube, à l’affût des phlébotomes. Elle tenait à emmener ses étudiants et à les confronter à la réalité et à la rigueur du terrain, convaincue qu’il s’agissait d’un préalable indispensable pour devenir un bon entomologiste et un bon épidémiologiste. Ses expéditions dans differentes régions du monde, riches en anecdotes, nourrissaient plus tard ses récits hauts en couleur, pimentés de son humour légendaire. Elle racontait notamment avec malice l’histoire de ce bédouin tombé sous son charme, qui demanda sans détour à son collègue d’expédition, le professeur Rioux, la permission de l’épouser. La vie de Nicole Léger, femme de terrain, fut remplie d’anecdotes, toutes plus croustillantes les unes que les autres. Dans les années 1990, le professeur Gentilini fut sollicité par le directeur de l’Opéra, affolé par l’état de santé des accessoiristes qui furent accablés par des démangeaisons contractées sur la scène, restées sans explication. Ils risquaient, menaçant de faire grève, de remettre en cause une représentation à la télévision, avec un budget de 60 millions de francs de l’époque dont avait absolument besoin l’Opéra pour son équilibre budgétaire. Le professeur Gentilini mobilisa Nicole Léger et elle procéda avec lui à une expertise locale qui aboutit à incriminer des acariens gîtés dans des gerbes de blé, pièces importantes du décor, ondulant sur le plateau et au milieu desquelles se démenaient les « petits rats » après les « gros bras ». De surcroît, chacun était inquiet pour la diva Montserrat Caballé. Ces gerbes furent jetées dans une benne au sein de la cour intérieure de l’Opéra pour être évacuées le lendemain matin. Mais, de nuit, des SDF s’en étaient emparés et les avaient revendues à bas prix dans le métro où un prurit inhabituel survint… Tout finit par s’arranger et le directeur de l’Opéra, heureux, offrit à Nicole Léger un abonnement qu’elle reçut avec une joie partagée. Universitaire dans l’âme, elle n’a jamais délaissé l’enseignement au profit de la recherche ou réciproquement. Durant les 12 années de l’existence du DEA Interactions Hôtes Parasites dirigé par le professeur René Houin, elle dirigea l’une des équipes constitutives de ce DEA et encadra nombre de stagiaires présentant des mémoires de grande qualité. Des années plus tard, certains rappelaient encore le souvenir exceptionnel et vivace qu’ils gardaient de ce stage. Dotée d’une culture générale exceptionnelle associée à un franc parler unique, elle a impressionné des générations d’étudiants auxquels elle a fait dans la grande majorité apprécier la parasitologie, ses cours étant ponctués d’anecdotes provenant de sa riche expérience de femme de terrain, masquant ainsi une exigence hors normes. Qui, ayant assisté à ses cours, ne se remémore pas ses envolées passionnées qui savaient maintenir en haleine tout un amphithéâtre? À la demande du doyen Vistelle, elle fut chargée au début des années 2000 de réaliser une formation continue en soirée. Le plus grand amphithéâtre des facultés de médecine et de pharmacie se révéla rapidement bien trop petit et plus d’une centaine de confrères assistèrent à son enseignement debout, ou mieux, assis sur les marches des escaliers. Elle se plaisait à compter nombre d’anecdotes qui, mises bout à bout, donnent un croquis assez descriptif de sa façon d’être et de penser. En voici quelques-unes. Ses trajets entre Paris et Reims en voiture de sport décapotable à vive allure, parfois sans ceinture de sécurité, l’ont amenée à devoir amadouer les motards de la police qui finirent par la laisser filer car attendue à l’hôpital pour le bien de tous. La fidélité des étudiants (moins les étudiantes) qui se plaçaient au premier rang de l’amphithéâtre lors de ses cours pour lesquels elle se hissait sur ses talons pour écrire en haut du tableau à une époque où la mode était aux jupes plutôt courtes. Sa détermination à obtenir des spécimens y compris dans des endroits difficilement accessibles. C’est ainsi que lors d’une mission de capture de phlébotomes dans des îles grecques, elle insista pour que deux de ses étudiants qui l’accompagnaient aillent poser des pièges dans une cavité se trouvant à flanc de paroi. Une fois parvenus non sans difficultés dans cette grotte qui amplifiait leur voix, ils se sont soulagés en exprimant ce qu’ils pensaient de l’entêtement de leur professeur qui, au bord de cette falaise, a donc pu aisément profiter de ces remarques peu avenantes. C’est après être remontés qu’ils ont soudainement acquis quelques notions supplémentaires de la physique du son.

Les conditions de sa découverte de l’hôte de *Leishmania guyanensis* sont dignes d’un roman. Alors en mission en Guyane avec un collègue botaniste du Muséum national d’Histoire naturelle, c’est en écoutant un prêtre lui faisant part d’une lésion *a priori* leishmanienne mal placée qu’elle lui a demandé s’il faisait une « pause technique » lors de ses aller-retours entre le village de Cacao et Cayenne. C’est ainsi que l’arbre qui lui servait de « vespasienne » à chaque trajet a été identifié et, par voie de conséquence, l’animal qui lui était associé à savoir le paresseux à deux doigts. Mais ces moments précieux, notamment chez elle autour de la table de cuisine, étaient aussi remplis d’histoires chargées d’émotion comme le récit qu’elle avait de sa grand-mère, vivant aux îles du Salut, et dont le précepteur avait été le capitaine Dreyfus. C’est autour de cette même table qu’elle a transmis avec force ce message à une jeune enfant: « Jamais peur! ».

**Figure 6:**
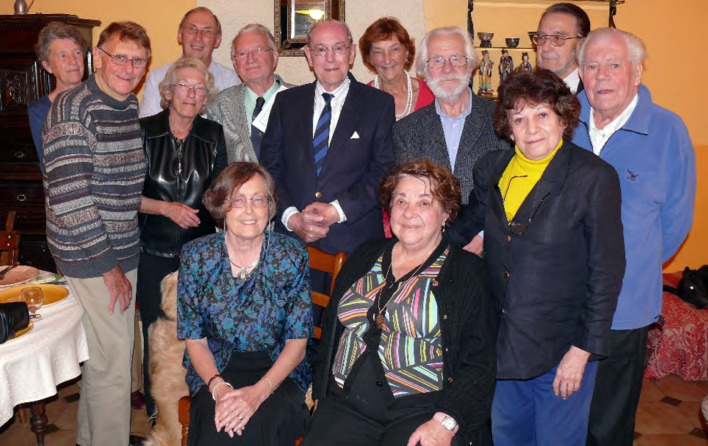
Photo prise par Bob Killick-Kendrick chez Alain Chabaud en 2007: réunion en 2007 de parasitologues anglais et français, amis de longue date (une indéfectible amitié les liait tous depuis des décennies). Debout: Odile Bain, John Baker (Cambridge), Irène Landau, Jean-Charles Gantier, Robert (dit Bill) Bray (Imperial College, Ascot, UK), Ralph Lainson (Belem, Brésil), Elizabeth Canning (Imperial College, Ascot, UK), Bob Killick-Kendrick (Imperial College, Ascot, UK), Jean-Antoine Rioux (Université de Montpellier), Alain Chabaud (MNHN), Zea Lainson. Assises: Mireille Killick-Kendrick et Nicole Léger (crédit photo: Bob Killick-Kendrick)
