# Supplementary material for: Tribute to Nicole Léger (1933-2024)
Source: Med Trop Sante Int. 2025 Jul 29;5(4):mtsi.v5i4.2025.728. [Article in French] doi: 10.48327/mtsi.v5i4.2025.728 (PMC12948357; doi:10.48327/mtsi.v5i4.2025.728)
Supplement: Related texts

# Research article: Tribute to Nicole Léger (1933-2024)

**Figure 1:** Portrait of Nicole Léger taken in the early 2000s (photo credit: Bob Killick-Kendrick)

Nicole Léger was born in 1933 and studied pharmacy at the University of Paris from 1950 to 1955. She completed her degree in 1956 and 1957, earning advanced certificates in biological chemistry, parasitology, serology, and bacteriology. After being resident at Paris hospitals in 1954 and psychiatric hospitals in 1958, she joined the Paris Faculty of Pharmacy in 1959 as an assistant teaching practical parasitology classes led by Professor Cavier. She then became head of practical work and senior assistant. She taught the special certificate in parasitology, first-year practical zoology, fourth-year tutorials, and fifth-year practical work. She did this while following the progress of the reform of pharmaceutical studies and setting up a new course each year. Simultaneously, she earned a Bachelor of Science degree and, in 1965, presented a doctoral thesis in pharmaceutical sciences entitled “Contribution to the study of the biological behavior of the cysticercoid of *Hymenolepis nana* var. *fraterna* vis-à-vis its hosts and drugs,” for which she received honors and the thesis prize in natural sciences. From 1963 onward, she worked as an assistant at the Assistance Publique. First, she worked at the Corentin Celton Hospital as a parasitology assistant. From 1968 onward, she worked in the department of Professor Marc Gentilini at the Saint-Louis Hospital, and then at the Pitié-Salpêtrière Hospital. She was received as associate professor in medicine, then associate professor in pharmacy. She was appointed professor in Reims in 1972 and ended her official career there in 2001. Due to her varied activities, her research, which initially focused on the pharmacodynamics of anthelmintics, gradually diversified. The guiding principle of her thesis was that helminths undergo a series of biological revolutions during their cycle, with each stage corresponding to different sensitivities to external agents, particularly drugs used in chemotherapy. Thus, these drugs may be completely ineffective at certain stages. The resistance of immature forms to anthelmintic treatments was already well-known. She demonstrated this with dichlorophen and mepacrine in the cysticercoid of *Hymenolepis nana* in both the direct cycle and the cycle involving an intermediate host. She also attempted to clarify the mode of action of various tenicides on the adult stage of the parasite. She studied the morphological changes in helminths under the influence of various tenicides and vermifuges *in vivo* in mice. This work enabled her to demonstrate that the actions of these substances were far from unambiguous, and she identified at least two different mechanisms.

Her study of the reactivity of the larval form of *Hymenolepis* in mealworm beetles led her to examine host-parasite relationships in the insect-cysticercoid system. The study of host-parasite interactions became a major scientific interest throughout her career. She focused on the conditions under which reactions to parasitism appear in insects and noted the absence of any visible phenomenon in species that are normally infectable by the oral route. However, insects refractory to this mode of infection could be parasitized by experimentally injecting hexacanth embryos hatched *in vitro* into their cavities. These insects reacted by encapsulating and melanizing the parasites. Nicole Léger believed that hemocyte cells played an essential role in this process. The hemocoel of insects that cannot be infected orally is therefore a suitable environment for the larval development of *Hymenolepis nana.* The next step was to determine where the barrier to contamination was located. Nicole Léger demonstrated that egg hatching occurred normally in the intestinal lumen, releasing a viable embryo. When this embryo was recovered from feces and injected into a new insect, it developed into a cysticercoid infectious to mice. After proposing the hypothesis that the peritrophic membrane constituted the barrier to the passage of the hexacanth embryo and its subsequent development in the general cavity, she concluded, based on her histological work, that destruction occurred immediately after crossing the intestinal epithelium. In collaboration with Professor Ambroise Thomas, Nicole Léger also studied the relationship between the adult worm and the definitive host.

**Figure 2:** Parasitology laboratory at the London School of Tropical Medicine and Hygiene showing, from left to right, Bob Killick-Kendrick in front of David Molyneux, Wallace Peters in white coat, Jean-Antoine Rioux and Nicole Léger, photo taken in the early 1970s (photo credit: LSTMH)

However, the direction in which Nicole Légers research progressed did not divert her initial interest in the pharmacodynamics of antiparasitic drugs. As part of her study of the relationship between chemical structure and pharmacodynamic activity, she performed tenicide tests on *Hymenolepis nana* var. *fraterna in vitro* and *in vivo* in mice, as well as vermifuge tests on *Syphacia obvelata, Aspiculuris* tetraptera (mouse pinworms), and *Nippostrongylus brasiliensis* (rat pinworms). Unfortunately, her attempt to use *Dipetalonema vitae* for filaricide testing was unsuccessful because this merion parasite, adapted to hamsters, was insensitive to the drugs used for human filariasis. Nevertheless, *Dipetalonema vitae* proved to be an effective model for studying the histopatho-genic action of filariae, which is partly immune in nature. She began collaborating with Professor Gentilini to study renal lesions observed during filariasis. The medical team was responsible for the clinical aspects, and Léger carried out the experimental work.

Maintaining the strains necessary for her experiments also allowed her to provide immunologists at the Parasitology Laboratory of Pitié-Salpêtrière University Hospital with material for routine work and research: *Dipetalonema vitae, Nippostrongylus brasiliensis, Trypanosoma lewisi,* and *Pneumocystis carinii.* She was the first person in France to obtain these parasites experimentally in rats.

She believed that with *Anthemosoma garnhami*, she had shown that therapeutic evidence could sometimes help systematists. This parasite was first isolated in 1967 from rodents of the genus *Acomys* and has since been maintained in mice. It is a non-pigmented intraerythrocytic protozoan. In 1969, it was described by Irène Landau, a professor at the National Museum of Natural History in Paris. The study of its morphology led Landau to classify the parasite as belonging to the Babesioideae superfamily within the Dactylosomidae family. Since all previously described species in this family are parasites of cold-blooded vertebrates (amphibians and reptiles), Nicole Léger wondered if this parasite might be a non-pigmented hematozoan, perhaps closer to *Plasmodium* than *Babesia.* She wanted to demonstrate to systematists the value of taking therapeutic trials into account more often since drugs active against *Babesia* are inactive against Plasmodium, and vice versa. Therefore, Nicole Léger undertook a study of the action of these two groups of substances on *A. garnhami.* The antimalarial drugs tested were chosen because they were particularly active against *Plasmodium* in mice: quinine, quinacrine, chloroquine, sulfadiazine, and chlortetracycline. However, none were active against *A. garnhami,* and the course of infection was the same in treated and control animals. In contrast, all of the anti-*Babesia* drugs tested (acaprine, acriflavine, and pentamidine) were effective. This result reinforced Irène Landau’s opinion of the validity of the new genus close to *Babesia* that she had created. Nevertheless, *Dactylosoma* and the Dactylosomids had to be reclassified as Coccidia.

Studying the mechanisms of action of antiparasitic drugs and host-parasite relationships in invertebrates gave Nicole Léger the opportunity to consider the relationships between parasites and their environment. Therefore, it was only natural that she became interested in parasitic ecology, especially since her training as a naturalist predisposed her to it. While training with field parasitologists, she conducted a study with her colleague, Claude Combes, from Perpignan, on the geographical distribution of batrachian trematodes. Initially, she investigated variations in the populations of parasitic helminths over time in *Rana temporaria* in a region covering Cerdagne and part of Andorra that had previously been surveyed. She qualitatively determined that the distribution of Digenea and Monogenea was stable. The only variations observed occurred in sites significantly altered by humans. This could be explained by the disruption of ecological conditions that had previously maintained populations of intermediate mollusk hosts and, to a lesser extent, frog populations.

The second region surveyed by Nicole Léger was the Camargue. In a biotope where green frogs and tree frogs cohabited, she observed four species of Digenea in *Hyla meridionalis* alone. This amphibian had previously been considered resistant to infection by these trematodes. She also studied the helminthological fauna of Corsican amphibians. Due to insular phenomena, the uniqueness of these amphibians raised fascinating questions of phylogeny and phylogeography.

In the 1970s, she conducted malacology research in Corsica and determined that the island was ready for the introduction of *Schistosoma haematobium.* In 2013, we learned that her prediction had come true. She continued her work on bilharzia and demonstrated its zoonotic nature caused by *Schistosoma mansoni* in Guadeloupe, showing that rats played a role in the parasitic cycle. Toward the end of her career, she collaborated with her former student, Hubert Ferté, on schistosomes during outbreaks of cercarial dermatitis caused by avian schistosomiasis in numerous French lakes.

However, it was the ecology of parasitic cycles involving insects that would ultimately capture her attention due to her previous work. Meeting Professor Alain Chabaud of the National Museum of Natural History in Paris was pivotal in her career, leading to their collaboration on a program to develop a model for studying the epidemiology of filariasis in the Camargue region.

In the late 1970s, when the first cases of airport malaria appeared, the Ministry of Health commissioned her to conduct entomological surveys in and around Paris’s international airports. She presented her findings at a symposium in Strasbourg in 1980, held to commemorate the centenary of Alphonse Laveran’s discovery of the malaria agent.

Another decisive meeting in Nicole Légers career was with Professor Jean-Antoine Rioux of Montpellier. As part of his epidemiological research on leishmaniasis in North Africa, Professor Rioux conducted a study on sand flies and their distribution in Corsica. Using proven, long-established trapping methods, she was able to make rapid progress. She primarily used non-attractive adhesive traps to assess the relative density of each species by recording the number of individuals captured per square meter of trap. In two years, she surveyed the entire island, exploring nearly 400 sites and collecting over 20,000 sand flies. Each fly was examined and identified. This work resulted in the first report of *Phlebotomus sergenti,* the primary vector of *Leishmania tropica.* With this inventory, she could form a fairly accurate picture of the potential for human leishmaniasis transmission in Corsica. *Phlebotomus perniciosus* appears to be the main vector because of its marked anthropophilia and high density in the phytogeographical zone covering the pubescent oak and holm oak series. This zone corresponds to the classic distribution of *Leishmania* endemic foci in southern mainland France.

**Figure 3:** Nicole Léger and Claude Combes, in the field at Lac des Bouillouses (Cerdagne) in 1970 (photo credit: Bernard Pesson)

She continued her fruitful collaboration with Professor Rioux by working in Morocco for five years, starting in 1972. She focused her work on the eco-epidemiology of leishmaniasis, a vector-borne disease for which studying vectors is essential. She devoted much of the second half of her career to this field. She worked in numerous leishmaniasis hotspots, primarily in the Mediterranean basin. Passionate about entomology, she became a leading figure in the international community of “leishmaniacs” and “sand fly lovers.” In the late 1980s, Professor Robert Killick-Kendrick began planning a conference focused solely on phlebotomine sand flies (ISOPS: International Symposium On Phlebotomine Sandflies). He decided that Italy should be the host country because the first phlebotomine, *Phlebotomuspapatasi,* was described there by Scopoli in 1786. He therefore asked Michele Maroli of the Istituto Superiore di Sanità in Rome to organize the conference there. Initially, Maroli was skeptical and wondered how many people would attend. Then, Nicole Léger, with her characteristic enthusiasm, offered her energetic support to organize the first ISOPS in 1991, collaborating with Maroli and Killick-Kendrick. The conference was a huge success and continues to this day. Nicole Léger never missed an ISOPS conference, except for the 2019 conference in the Galápagos Islands, which she was unable to attend due to serious health concerns. She organized the 2016 conference in Reims, France. A vibrant tribute was paid to her at the last conference in September 2024 in Slovenia.

Passionate about taxonomy and systematics, she described 36 new taxa of phlebotomine sand flies: one tribe, two sub-tribes, three subgenera, and 30 species and subspecies.

**Figure 4:** Nicole Léger in the field in Rhodes in 1992 (photo credit: Jérôme Depaquit)

**Figure 5:** Nicole Léger at the center of participants at the first “International Symposium On *Phlebotomine* Sandflies” in Rome, in 1991 (photo credit: ISOPS)

## Taxa described in sand flies by Nicole Léger

**Tribe**

Hertigiini Abonnenc & Léger, 1976

**Subtribes**

Hertigiina Abonnenc & Léger, 1976

Lutzomyiina Abonnenc & Léger, 1976

Subgenera:

*Vattieromyia* Depaquit, Léger & Robert, 2008

*Trouilletomyia* Depaquit & Léger, 2014

*Madaphlebotomus* Depaquit, Léger & Randrianambinintsoa, 2015

**Species and subspecies**

*Phlebotomus (Larroussius) mariae* Rioux, Croset, Léger & Bailly-Choumara, 1974

*Phlebotomus (Madaphlebotomus) berentiensis* (Léger & Rodhain, 1978)

*Nyssomyia pajoti* (Abonnenc, Léger & Fauran 1979)

*Psychodopygus claustrei* (Abonnenc, Léger & Fauran, 1979)

*Nyssomyia bibinae* (Léger & Abonnenc, 1988)

*Australophlebotomus maduloae* Léger & Pesson, 1993

*Australophlebotomus notteghemae* Léger & Pesson, 1993

*Idiophlebotomus boucheti* (Léger & Pesson, 1994)

*Oligodontomyia isopsi* (Léger & Ferté, 1996)

*Phlebotomus* (*Synphlebotomus*) *saltiae* Léger, Haddad & Chaker, 1997

*Phlebotomus* (*Paraphlebotomus*) *riouxi* Depaquit, Ferté & Léger, 1998

*Phlebotomus* (*Transphlebotomus*) *economidesi* Léger, Depaquit & Ferté, 2000

*Phlebotomus (Madaphlebotomus) fertei* (Depaquit, Léger & Robert, 2002)

*Sergentomyia (Trouilletomyia) huberti* Depaquit, Léger & Robert, 2002

*Phlebotomus* (*Madaphlebotomus*) *fontenillei* (Depaquit, Léger & Robert, 2004)

*Chinius barbazani* Depaquit, Léger & Beales, 2006

*Chinius eunicegalatiae* Depaquit, Léger & Beales, 2006

*Phlebotomus (Euphlebotomus) mascomai* Müller, Depaquit & Léger, 2007

*Sergentomyia majungaensis* Depaquit, Léger & Robert, 2007

*Sergentomyia (Vattieromyia) anka* Depaquit, Léger & Robert, 2008

*Sergentomyia (Vattieromyia) namo* Depaquit, Léger & Robert, 2008

*Sergentomyia (Vattieromyia) sclerosiphon* Depaquit, Léger & Robert, 2008

*Phlebotomus (Euphlebotomus) barguesae* Depaquit, Muller & Léger, 2009

*Chinius samarensis* Léger, Depaquit & Gay, 2012

*Sergentomyia (Vattieromyia) pessoni* Depaquit, Randrianambinintsoa & Léger, 2012

*Sergentomyia (Rondanomyia) goodmani co-morensis* Depaquit, Randrianambinintsoa & Léger, 2012

*Phlebotomus* (*Madaphlebotomus*) *vaomalalae* (Randrianambinintsoa, Léger, Robert & Depaquit, 2013)

*Idiophlebotomus padillarum* Léger, Depaquit & Gay, 2014

*Sergentomyia brunhesi* Léger, Randrianambinintsoa & Depaquit, 2020

*Sergentomyia vistellei* Depaquit, Randrianambisintsoa & Léger, 2020

In the field of systematics, Nicole Léger greatly admired the work of her Brazilian colleague, Eunice Galati. The two formed a deep friendship. Along with Philippe Rispail, she began the phylogenetic analysis of Old World sand flies by studying morphological characteristics. She pioneered the supervision of Jérôme Depaquit and Guillaume Lecointre’s first molecular systematics work on phlebotomine sand flies. With her contagious enthusiasm, she trained many students and worked in many parts of the world. She was particularly fond of Greece, where she conducted a study on the speciation of sand flies based on insularity. An indefatigable traveler and proponent of excellence in fieldwork, she participated in and led numerous missions throughout the Mediterranean basin (Corsica, Spain, Italy, Malta, Serbia, Montenegro, Greece, Cyprus, Syria, Lebanon, and Morocco), the Canary Islands, and Africa (Benin, Cameroon, the Central African Republic, Namibia, South Africa, and Tanzania), and India

While studying phlebotomine sand flies, she met Émile Abonnenc, the French expert on these insects. She became one of his students and owed him a huge debt of gratitude. She had fond memories of not only her mentor, but also the loyal friend he had become over the years. She recognized him as a hardworking, meticulous man endowed with great honesty, rare kindness, and total selflessness. In French Guiana, she worked with Abonnenc on a massive project to compile information on American sand flies. She had become so close to Abonnenc that he left her his collection of sand flies. Her inherited collection and her own are now at the Faculty of Pharmacy in Reims, where they can be examined or loaned to the research community, as she wished.

Throughout her career, she cultivated an impressive network of collaborators around the world, including in France. Her students still benefit from this network today.

As a sign of her peers’ widespread recognition, six taxa have been named after her, including one subgenus and five species.

## Taxa named after Nicole Léger

**Insect**

Diptera, Keratoplatidae:

*Xenoplatyura nicolae* Matile, 1997

Diptera, Psychodidae:

*Lutzomyia (Tricholateralis) legerae* Le Pont, Gantier, Hue & Valle, 1995

Sous-genre *(Legeromyia)* Rahola, Depaquit, Makanga & Paupy, 2013

*Idiophlebotomus nicolegerae* Loyer, Depaquit & Gay, 2016

**Nematods**

Nematoda, Molineidae:

*Molineus legerae* Durette-Desset & Pesson, 1987

Nematoda, Capillariidae:

*Capillaria legerae* Justine, Ferté & Bain, 1987

Nicole Légers scientific work appears quite diverse. However, her underlying motivations can be defined by her desire to understand the factors that regulate the emergence and maintenance of parasitism, whether in the limited context of the host’s internal environment or in the broader context of biocenoses.

At a time when strict specialization had become a necessity for progress in most sciences, she had probably chosen one of the last disciplines in which eclectic knowledge and curiosity retained their full value. Parasites are strictly dependent on their environment, so their study requires the ability to be a zoologist, botanist, histologist, immunologist, ecologist, or even a chemist at times. In recent decades, aspiring naturalists have often been misunderstood. However, it seems that humans have finally realized that they cannot cut themselves off from nature, of which they are an integral part, without consequence. Current events particularly vindicate Nicole Léger, as rehabilitation is finally underway. Today, who does not subscribe to an approach that integrates human, animal, and environmental health under the concept of “one health”? She was a pioneer in this field, as in so many others.

Throughout her life and career, Nicole Léger was fortunate to have her husband Pierre’s encouragement, help, and active support. Pierre was a renowned surgeon. He often accompanied her to conferences and field missions, participating in her work and making significant contributions to her studies.

Always ready for adventure, Léger did not hesitate to sleep under the stars in uncomfortable conditions so she could look for sand flies at dawn. She was eager to bring her students along and expose them to the realities and challenges of fieldwork, believing it was essential for becoming good entomologists and epidemiologists. Her colorful stories, spiced with her legendary humor, were fed by her expeditions to different parts of the world, rich in anecdotes. She mischievously recounted the story of a Bedouin who fell under her spell and asked her expedition colleague, Professor Rioux, point-blank for permission to marry her. The life of Nicole Léger, a woman of action, was full of colorful anecdotes. In the 1990s, Professor Gentilini was called upon by the director of the opera house, who was distraught over the health of the prop masters. They were suffering from an itchy rash that they had contracted on stage. The prop masters threatened to go on strike, which would have jeopardized a television performance with a budget of 60 million francs at the time. The opera house desperately needed this performance to balance its books. Professor Gentilini enlisted Nicole Léger to help him conduct a local investigation that revealed mites in the wheat sheaves. The wheat sheaves were an important part of the set and swayed on stage, allowing the “little opera rats” to wriggle among them after the “big guns.” Everyone was also worried about the diva Montserrat Caballé. The sheaves were thrown into a dumpster in the inner courtyard of the opera house, scheduled for removal the next morning. However, during the night, homeless people took them and sold them at low prices in the subway. An unusual itching broke out there. In the end, everything worked out, and the director of the opera delighted Nicole Léger by offering her a season ticket, which she accepted with shared joy. An academic at heart, she never chose research over teaching or vice versa. During the twelve years of the Host-Parasite Interactions DEA (postgraduate diploma), which was directed by Professor René Houin, she led one of the DEA teams and supervised interns who produced high-quality theses. Years later, her former interns still recalled their exceptional and vivid memories of the program. With her exceptional general knowledge and unique frankness, she impressed and inspired generations of students to appreciate parasitology. Her lectures were punctuated with anecdotes from her rich experience as a woman in the field and masked her exceptionally high standards. Who among those who attended her lectures can forget her passionate outbursts that kept the entire lecture hall spellbound? At the request of Dean Vistelle, she set up an evening continuing education program in the early 2000s. The largest lecture hall in the medical and pharmacy faculties quickly proved to be far too small, with more than a hundred colleagues attending her classes standing up or sitting on the stairs. She enjoyed recounting numerous anecdotes that, when pieced together, paint a fairly descriptive picture of her personality and way of thinking. Here are a few. She would take trips between Paris and Reims in a fast convertible sports car, sometimes without a seatbelt. This led her to have to appease police motorcyclists, who eventually let her go because she was needed at the hospital. There was the loyalty of her students (except the female students), who sat in the front row of the lecture hall during her classes. She would stand on her tiptoes to write at the top of the blackboard, at a time when short skirts were in fashion. She was determined to obtain specimens, even in diffi-cult-to-access places. During a mission to capture sand flies on the Greek islands, she insisted that two students accompanying her set traps in a cavity in a cliff. Once they had reached the cave, which amplified their voices, they vented their frustration at their professor’s stubbornness, who, standing on the edge of the cliff, was able to hear their unflattering remarks. It was only after they had climbed back up that they suddenly gained some additional insight into the physics of sound. The circumstances surrounding her discovery of the *Leishmania guyanensis* host are worthy of a novel. While on assignment in French Guiana with a botanist from the National Museum of Natural History, she listened to a priest describe a presumably leishmaniasis lesion in an unusual location. She then asked the priest if he took “technical breaks” during his trips between the village of Cacao and Cayenne. This led to the identification of the tree he used as a “restroom” on each trip, as well as the animal associated with it: the two-toed sloth. These precious moments, especially at her kitchen table, were also filled with emotionally charged stories. One such story was about her grandmother, who lived on the Îles du Salut and whose tutor had been Captain Dreyfus. It was around this same table that she conveyed this powerful message to a young child: “Never be afraid!”

**Figure 6:** Photo taken by Bob Killick-Kendrick at Alain Chabaud’s home in 2007: 2007 meeting of English and French parasitologists, long-time friends (an unwavering friendship had bound them all together for decades). Standing: Odile Bain, John Baker (Cambridge), Irène Landau, Jean-Charles Gantier, Robert (known as Bill) Bray (Imperial College, Ascot, UK), Ralph Lainson (Belem, Brazil), Elizabeth Canning (Imperial College, Ascot, UK), Bob Killick-Kendrick (Imperial College, Ascot, UK), Jean-Antoine Rioux (University of Montpellier), Alain Chabaud (MNHN), Zea Lainson. Seated: Mireille Killick-Kendrick and Nicole Léger (photo credit: Bob Killick-Kendrick)
